# Anti-Acne Effects of Cembrene Diterpenoids from the Cultured Soft Coral *Sinularia flexibilis*

**DOI:** 10.3390/md18100487

**Published:** 2020-09-25

**Authors:** Li-Wei Chen, Hsuan-Lien Chung, Ching-Chiung Wang, Jui-Hsin Su, Yu-Ju Chen, Chia-Jung Lee

**Affiliations:** 1 Program in Clinical Drug Development of Herbal Medicine, College of Pharmacy, Taipei Medical University, Taipei 11031, Taiwan; serochen@cgmh.org.tw (L.-W.C.); crystal@tmu.edu.tw (C.-C.W.); yjchen72@tmu.edu.tw (Y.-J.C.); 2 Department of Chinese Herbal Pharmacy, Taoyuan Chang Gung Memorial Hospital, Taoyuan 33378, Taiwan; 3 Graduate Institute of Pharmacognosy Science, College of Pharmacy, Taipei Medical University, Taipei 11042, Taiwan; zzz810355@gmail.com; 4 School of Pharmacy, Taipei Medical University, Taipei 11031, Taiwan; 5 Traditional Herbal Medicine Research Center, Taipei Medical University Hospital, Taipei 11042, Taiwan; 6 National Museum of Marine Biology and Aquarium, Pingtung 94450, Taiwan; x2219@nmmba.gov.tw; 7 Graduate Institute of Marine Biology, National Dong Hwa University, Pingtung 94450, Taiwan

**Keywords:** acne, *Sinularia flexibilis*, cembrene diterpenoids, *Cutibacterium acnes*, inflammation, MAPK

## Abstract

Acne is a skin disease common in adolescents and increasingly common in the adult population. The major pathologic events of acne vulgaris include increased sebum production, retention hyperkeratosis, carrying commensal skin microbiota, and inflammation. In recent years, more than 10,000 compounds have been isolated and identified from marine organisms. The aim of this study was to discover the potential anti-acne activity of fraction 9 + 10 (SF-E) of *Sinularia flexibilis* extract and six cembrene diterpenoids. We found that the SF-E significantly reduced *Cutibacterium acnes*-induced edema in Wistar rat ears. The cembrene diterpenoids including 11-dehydrosinulariolide (SC-2), 3,4:8,11-bisepoxy-7-acetoxycembra-15(17)-en-1,12-olide (SC-7), and sinularin (SC-9) reduced nitric oxide (NO) production with 50% inhibitory concentration of 5.66 ± 0.19, 15.25 ± 0.25, and 3.85 ± 0.25 μM, respectively, and inducible NO synthase expression in RAW 264.7 cells. Moreover, treatment with SC-2, SC-7, and SC-9 significantly suppressed lipopolysaccharide- and heat-killed *C. acnes*-induced expression of proteins involved in mitogen-activated protein kinase pathway in both RAW 264.7 and HaCaT cells. After treatment with SC-2, SC-7, and SC-9, over-proliferation of HaCaT cells was significantly terminated. In summary, SC-2, SC-7, and SC-9 showed anti-inflammatory effects in RAW 264.7 cells, suggesting that these cembrene diterpenoids obtained from *S. flexibilis* are natural marine products with potential anti-acne activities.

## 1. Introduction

Acne, also known as acne vulgaris, is a skin disease that usually appears in young adolescents with a hormone imbalance, and increasingly common in the adult population such as polycystic ovary syndrome (PCOS) patients. It can be a significant risk factor for psychological morbidity at all ages in both sexes [1]. In addition, acne is associated with decreased self-esteem and self-confidence, and increased internalizing problems (anxiety and depression) and suicidal ideation. Therefore, it is necessary to develop effective acne treatment drugs [2]. There are four major causes of acne formation: (1) increased sebum production by overactive oil glands, (2) retention hyperkeratosis, which blocks skin pores, (3) activities leading to carrying of commensal skin microbiota (*Cutibacterium acnes* and *Staphylococcus aureus*), and (4) skin inflammation [3]. Hormonal activity, associated with puberty and menstrual cycles, may aggravate acne formation. During puberty, increases in androgen hormones such as testosterone and dihydrotestosterone (DHT) can cause skin follicle glands to enlarge and produce more oil sebum [4]. The anaerobic bacterial species *C. acnes* is the major pathogenic bacteria of acne. *C. acnes* has the capabilities to change, perpetuate, and adapt to the abnormal cycle of inflammation, oil production, and inadequate sloughing of dead skin cells from acne pores. Isotretinoin is an extremely effective drug, inhibiting all four pathogenic mechanisms but with a teratogenic effect. Furthermore, various therapeutic agents such as azelaic acid result in skin depigmentation and retinoic acid and salicylic acid also lead to skin irritation and desquamation [3,5]. Treatment of acne vulgaris with antibiotics, including oral tetracycline and topical erythromycin and clindamycin, has increased the drug resistance of *C. acnes* to these antibiotics. Normal sebocytes secrete a small amount of IL-1α, TNF-α, IL-6, and IL-8, but when stimulated by *C. acnes* or lipopolysaccharide (LPS), the secretion of these cytokines is enhanced significantly. In addition, *C. acnes* can induce the inflammatory response of macrophages or monocytes, resulting in secretion of proinflammatory cytokines. According to previous reports, these proinflammatory cytokines can cause the appearance of skin adhesion molecules and chemotaxis of inflammatory cells that attract the neutrophils into the sebaceous unit to produce a subsequent inflammatory response [6,7,8]. Nonsteroidal anti-inflammatory drugs (NSAIDs), such as ibuprofen, diclofenac, and mefenamic acid, are typically used to inhibit inflammatory reactions, but the common side effects of NSAIDs are peptic ulcers and liver toxicity [9].

Diterpenes from soft corals have been reported to display different bioactive properties such as anti-inflammatory, antibacterial, antiviral, and anti-cancer [10]. Cembrene diterpenoids have been frequently reported from the soft coral *Sinularia flexibilis*, many of which carrying biological properties. Sinulariolide, an active compound isolated from *S. flexibilis,* inhibits the proliferation and migration of A375 melanoma cells, through a pathway that induces apoptosis through caspase cascade activation and mitochondrial dysfunction [11]. 11-Episinulariolide acetate (Ya-s11), a cembrene-type compound from a Formosan soft coral *Sinularia querciformis,* inhibits the expression of proinflammatory proteins such as induced nitric oxide synthase (iNOS) and cyclooxygenase (COX)-2 in LPS-stimulated murine macrophages [12].

Owing to the multiple factors involved in the pathogenesis of acne, a single-effect drug development strategy is suboptimal. Finding a drug that exerts multiple therapeutic effects will be beneficial to treat acne. Moreover, anti-acne effects of marine resources have not been well reported. In addition, marine soft corals have significant anti-inflammatory and bacterial growth-inhibiting activities. Therefore, we aimed to evaluate the anti-inflammatory and bacterial growth-inhibiting activities, and inhibition of DHT-induced proliferation of HaCaT cells of cembrene diterpenoids from *S. flexibilis*. Moreover, Moreover, the structure activity relationship (SAR) of anti-acne effects of cembrene diterpenoids from *S. flexibilis* are also discussed.

## 2. Results

### 2.1. S. flexibilis Attenuated C. acnes-Induced Ear Edema in Wistar Rats

**Figure 1:**
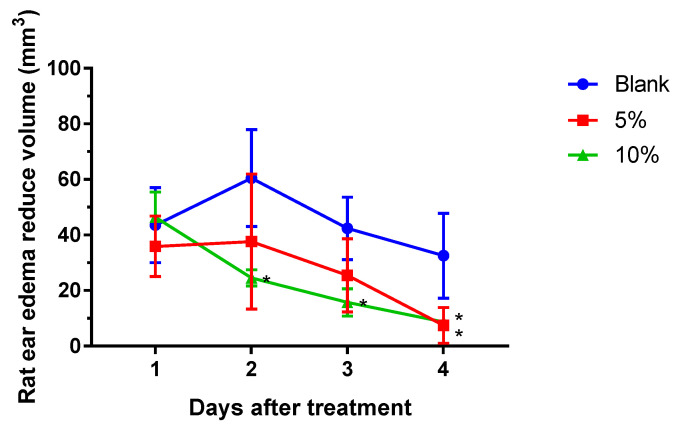
Anti-acne effects of SF-E ointment against *Cutibacterium acnes* injection in rats’ ears. * *p* < 0.05, compared to the blank group. Data are presented as the mean and standard deviation. Each group contained five rats.

*C. acnes* was directly injected into rats’ ears to cause acne formation, and we evaluated the in vivo anti-acne effects of the SF-E. Live *C. acnes* was injected into Wistar rats’ ears, and persistent edema occurred for 4 days (Figure 1). In the blank group, rat ear edema continued increasing up to the second day and slightly decreased on the third and fourth day post-injection. In the SF-E ointment groups, both dosages (5% and 10%) significantly reduced rat ear edema compared with that in the vehicle blank group.

### 2.2. S. flexibilis and Cembrene Diterpenoids Did Not Attenuate C. acnes Growth

The anaerobic bacterial species *C. acnes* contributes to the development of and is associated with moderate and severe inflammatory acne. Therefore, *C. acnes* was used to explore the anti-acne activity. We observed that the diameters of the inhibition zones of SF-E and cembrene diterpenoids were not significantly clear. Hence, the SF-E and six cembrene diterpenoids had no antibacterial effect on *C. acnes* (data not shown).

### 2.3. Cembrene Diterpenoids Displayed an Anti-Inflammatory Effect in LPS-Induced RAW 264.7 Cells

The inhibitory effects of six cembrene diterpenoids (SC-2, SC-4, SC-6, SC-7, SC-9, and SC-10) on NO were measured in LPS-treated RAW 264.7 cells for 24 h. NO levels were detected in the culture medium using the Griess reaction. Survival rates were detected by an MTT assay. The six cembrene diterpenoids presented no cytotoxicity at 20 µM (Table 1). Compounds SC-2, SC-7, and SC-9 displayed more potent NO inhibition effects than the others, with 50% inhibitory concentration (IC_50_) values of 5.66 ± 0.19, 15.25 ± 0.25, and 3.85 ± 0.25 µM, respectively. However, SC-2, SC-7, and SC-9 had no inhibitory effect on prostaglandin E_2_ (PGE_2_) production at 24 h. Furthermore, SC-9 presented greater PGE_2_ inhibitory effects than that of others after treatment with LPS for 6 h, with an IC_50_ value of 6.22 µM.

### 2.4. Effects of SC-2, SC-7, and SC-9 on Protein Expressions of Phosphorylated Extracellular Signal-Regulated Kinase (p-ERK), Phosphorylated c-Jun N-Terminal Kinase (p-JNK), iNOS, and COX-2 in LPS-Induced RAW 264.7 Cells

**Figure 2:**
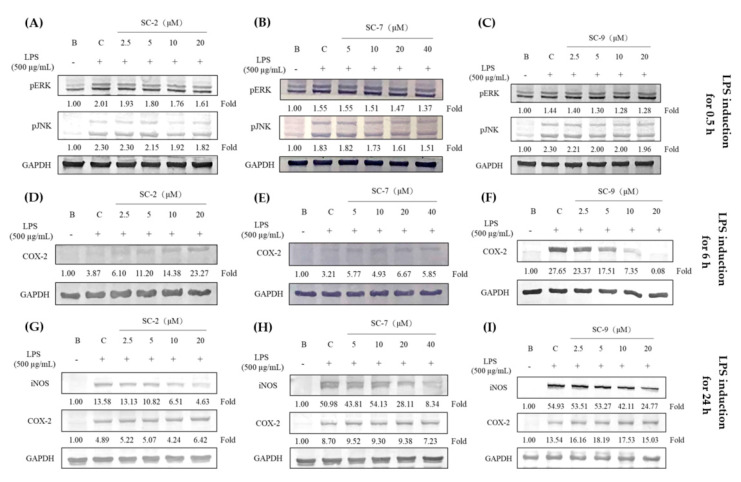
Effects of SC-2, SC-7, and SC-9 against lipopolysaccharide (LPS)-induced inflammation in RAW 264.7 cells, and its correlation with protein expression of phosphorylated extracellular signal-regulated kinase (p-ERK) and phosphorylated c-Jun N-terminal kinase (p-JNK) at 0.5 h (**A**–**C**), cyclooxygenase (COX)-2 at 6 h (**D**–**F**), and inducible nitric oxide synthase (iNOS) and COX-2 at 24 h after LPS induction (**G**–**I**). GAPDH: glyceraldehyde-3-phosphate dehydrogenase.

Based on the data above, expression of inflammation-associated proteins was analyzed. We explored the inhibitory effects of SC-2, SC-7, and SC-9 in LPS-induced RAW 264.7 cells for 0.5, 6, and 24 h. SC-2 (2.5–20 μM), SC-7 (5–40 μM), and SC-9 (2.5–20 μM) displayed dose-dependent inhibitory effects on p-ERK and p-JNK in the MAPK pathway after 30 min of induction (Figure 2A–C). Compound SC-9 expressed more significant COX-2 inhibitory effects at 6 h than that at 24 h (Figure 2D–F). Furthermore, SC-2 and SC-7 did not present identical COX-2 inhibitory effects. Compounds SC-2, SC-7, and SC-9 decreased iNOS protein expression at 24 h (Figure 2G–I). Taken together, SC-2, SC-7, and SC-9 decreased iNOS and COX-2 expression by inhibiting p-ERK and p-JNK in the MAPK pathway in LPS-induced RAW 264.7 cells with SC-9 being the strongest among them.

### 2.5. Cembrene Diterpenoids Displayed Anti-Inflammatory Effects in Heat-Killed C. acnes-Treated RAW 264.7 Cells

**Figure 3:**
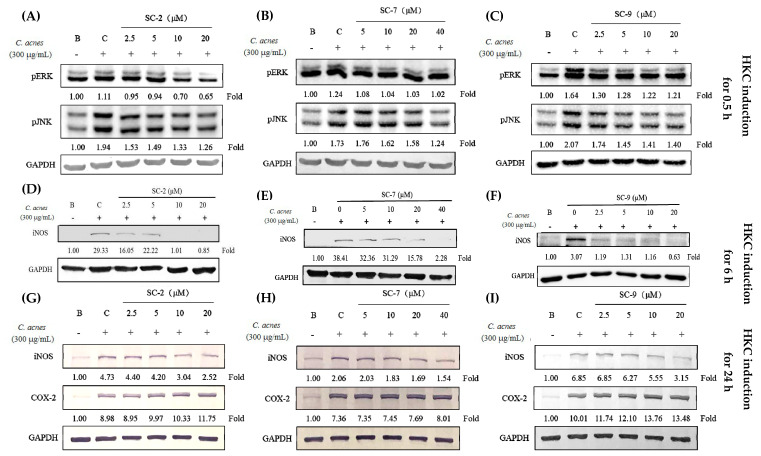
Effects of SC-2, SC-7, and SC-9 on inflammation in heat-killed *Cutibacterium acnes* (HKC)-treated RAW 264.7 cells, and its correlation with protein expression of phosphorylated extracellular signal-regulated kinase (p-ERK), phosphorylated c-Jun N-terminal kinase (p-JNK) at 0.5 h (**A**–**C**), inducible nitric oxide synthase (iNOS) at 6 h (**D**–**F**), and iNOS and cyclooxygenase (COX-2) at 24 h after HKC induction (**G**–**I**). GAPDH: glyceraldehyde-3-phosphate dehydrogenase.

We also used heat-killed *C. acnes* (HKC) to simulate the irritation and inflammation caused by *C. acnes* infection. SC-2, SC-7, and SC-9 inhibited NO production in HKC-treated RAW 264.7 cells with IC_50_ values of 5.81 ± 0.4, 7.42 ± 0.4, and 2.81 ± 0.2 μM, respectively (Table 1). Furthermore, SC-2 (2.5–20 μM), SC-7 (5–40 μM), and SC-9 (2.5–20 μM) showed good inhibitory effects on p-ERK and p-JNK expression in the MAPK pathway. Although SC-2, SC-7, and SC-9 showed significant inhibitory effects on iNOS expression, no changes were found in COX-2 expression (Figure 3).

### 2.6. SC-2, SC-7, and SC-9 Displayed Anti-Inflammation in Heat-Killed C. acnes-Treated HaCaT Cells

**Figure 4:**
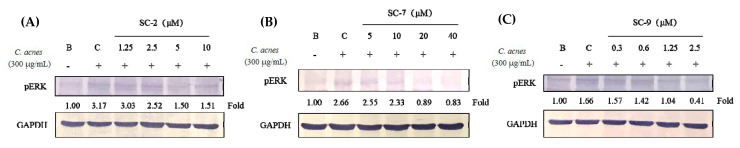
Effects of SC-2 (**A**), SC-7 (**B**), and SC-9 (**C**) on phosphorylated extracellular signal-regulated kinase (p-ERK) protein expression in heat-killed *Cutibacterium acnes* (HKC)-treated HaCaT cells at 30 min after HKC induction. GAPDH: glyceraldehyde-3-phosphate dehydrogenase.

We further used heat-killed *C. acnes* to induce human normal skin keratinocyte (HaCaT cell) inflammation and explore the effects of various factors on keratinocytes. Cytotoxicity analysis showed that SC-2, SC-7, and SC-9 presented no cytotoxicity to HaCaT cells at 1.25–10, 5–40, and 0.312–2.5 μM, respectively. In addition, SC-2 and SC-9 caused growth inhibition of HaCaT cells at high doses, indicating effects of inhibiting keratinogenesis. Compounds SC-2, SC-7, and SC-9 showed significant inhibitory effects on p-ERK (Figure 4). However, NO production and iNOS expression were not induced in HKC-treated HaCaT cells (data not shown).

### 2.7. SC-2, SC-7, and SC-9 Displayed Anti-Proliferative Effects in Testosterone- and DHT-Treated HaCaT Cells

As keratinocyte over-proliferation is a crucial event in acne formation, testosterone and DHT were used as inducers to stimulate HaCaT cell proliferation. We observed that 20 nM of testosterone and DHT significantly induced HaCaT cell proliferation at 48 h. The DHT concentrations of 0.1, 0.5, 1, and 5 μg/mL resulted in HaCaT cell viability of 97.6%, 103.2%, 104.7%, and 118.1%, respectively. Compounds SC-2, SC-7, and SC-9 significantly decreased testosterone-induced HaCaT cell proliferation at concentrations of 1.25–5, 2.5–10, and 0.08–0.32 μM, respectively (Table 2).

## 3. Discussion

So far, more than 10,000 compounds have been isolated and identified from marine organisms [13], among which, corals and sponges rank as the most prolific sources. Most previous studies on natural compounds from soft corals have reported anti-inflammatory, analgesic, and anti-neuritis properties [14,15]. Since the efficacy of these compounds in treating acne has not been evaluated well, this study attempted to discover their potential anti-acne activities. This study was mainly designed based on the causes of acne formation and explored the mechanisms of cembrene diterpenoids on the inflammatory response and sebum secretion.

Herein, live *C. acnes* was injected into Wistar rat ears to produce inflammatory effects such as edema to analyze the anti-acne effects of the SF-E, which containing 50% of SC-2, SC-7, and SC-9 in vivo. We found that SF-E ointment could inhibit the inflammatory response caused by the growth of *C. acnes* and reduce ear edema. However, in terms of antibacterial effects, the SF-E and six cembrene diterpenoids were unable to inhibit *C. acnes* growth (data not shown). These findings suggested that cembrene diterpenoids from *S. flexibilis* do not directly inhibit bacterial growth but use another pathway to reduce bacteria-induced acne effectively without antibiotic resistance.

Therefore, LPS and HKC were used as inducers to analyze the anti-inflammatory effects of these cembrene diterpenoids. LPS, a component of the outer membrane of gram-negative bacteria, is the most commonly known inflammation inducer [16]. RAW 264.7 cells were stimulated with LPS through toll-like receptor 4 (TLR4) to upregulate the MAPK and nuclear factor (NF)-κB pathways to express iNOS and COX-2 and produce NO and PGE_2_ [17]. Furthermore, LPS also mediates myeloid differentiation factor 88 (MyD88), which activates NF-κB and activating protein (AP)-1 and produces proinflammatory factors such as TNF-α and IL-6 [18]. However, *C. acnes*, a gram-positive bacterium, stimulates macrophages to produce JNK/ERK and AP-1/NF-κB through toll-like receptor 2 (TLR2) [19]. We observed that both LPS and HKC induced iNOS/NO and COX-2/PGE_2_ via the JNK/ERK pathway (Figure 2 and Figure 3). SC-2, SC-7, and SC-9, cembrene diterpenoids isolated from *S. flexibilis*, showed significant inhibitory effects on inflammation caused by both gram-positive and gram-negative bacteria.

We used RAW 264.7 cells and HaCaT keratinocytes for evaluating the anti-inflammatory effects. Macrophages activate various cytokines and inflammatory mediators such as NO, IL-6, and TNF-α, which in turn activate a skin inflammatory response [20]. HKC induced TLR2/MAPK protein expression in HaCaT keratinocytes and iNOS, p-ERK, and p-JNK expression in the inflammatory pathway to produce the proinflammatory factors NO and PGE_2_ in RAW 264.7 cells. Moreover, HaCaT keratinocytes only induce the expression of p-ERK and not NO or iNOS [21], indicating that different inflammatory response pathways may be involved. However, both macrophages and keratinocytes can cause severe skin inflammation under specific conditions. Our findings showed that SC-2, SC-7, and SC-9 inhibited expression of p-ERK in the MAPK pathway to achieve anti-inflammatory effects in both RAW 264.7 cells and HaCaT keratinocytes.

Furthermore, SC-2, SC-7, and SC-9 inhibited proinflammatory cytokines and reduced the expression of inflammation pathway-related proteins. Comparing SC-2 and SC-4 structures, we found that the heptalactone ring of SC-4 on carbon 1 was ring-opened, leading to a loss of anti-inflammatory functions. The same phenomenon was observed for SC-6 and SC-7. The structure of SC-9 was ring-opened, which eliminated the anti-inflammatory activity. Comparing SC-9 and SC-10, SC-10 was found to be hydrogenated on carbon 17 forming a single bond, and its anti-inflammatory effects were less than that of SC-9. Therefore, SC-4, SC-6, and SC-10 showed no inhibitory effects on NO expression. This proves that the heptalactone ring and double bond on carbon 17 are active functional groups. SC-9 presented the best NO and PGE_2_ inhibitory activities through inhibiting long-term iNOS and short-term COX-2 protein expression.

Lactone rings are widely found in nature, such as ascorbic acid, antibiotics (erythromycin and amphotericin B), anti-cancer drugs (vernolepin and epothilone). Caprolactone is a lactone (a cyclic ester) possessing a seven-membered ring. Alkali or heating can hydrolyze the lactone into a bifunctional compound. Soft corals have been known to be the organisms possessing secondary metabolites with high diversity in chemical structures. flexibilisolides C, flexibilisolides D, 11-dehydrosinulariolide, flexilarin D, 11-epi-sinulariolide acetate, 26 3,4:8,11-bisepoxy-7-acetoxycembra-15(17)-en-1,12-olide (SC-7), 27 sinulariolide, and 11-epi-sinulariolide could significantly inhibit the accumulation of the proinflammatory iNOS protein and flexibilisolides C, 11-dehydrosinulariolide, 11-epi-sinulariolide acetate, and 11-epi-sinulariolide could reduce the accumulation of COX-2 protein in LPS-stimulated RAW264.7 macrophage cells [22]. However, the SAR was not discussed, nor was any discussion on open ring. Therefore, this paper is the first to discuss the structure and anti-inflammatory activity. In the future, we will continue to separate related components for further analysis and evaluation.

## 4. Materials and Methods

### 4.1. Materials

Six components of *S. flexibilis* including, 11-dehydrosinulariolide (SC-2), sinulariolide (SC-4), dendronpholide F (SC-6), 3,4:8,11-bisepoxy-7-acetoxycembra-15(17)-en-1,12-olide (SC-7), sinularin (SC-9), and dihydrosinularin (SC-10), were isolated and identified by Prof. Jui-Hsin Su (National Museum of Marine Biology and Aquarium, Pintung, Taiwan) [23]. Dimethyl sulfoxide (DMSO), LPS, 3-(4,5-dimethylthiazol-2-yl)-2,5-diphenyltetrazolium bromide (MTT), napthyl-ethylene-dihydrochloride, sulfanilamide, and other chemicals were purchased from Sigma-Aldrich (St. Louis, MO, USA). RAW 264.7 (BCRC 60001) and HaCaT cells were purchased from Bioresource Collection and Research Center (Hsinchu, Taiwan). Dulbecco’s Modified Eagle Medium (DMEM), fetal bovine serum (FBS), antibiotics, and glutamine were purchased from GIBCO BRL (Grand Island, NY, USA). The bacterial culture equipment, including an anaerobic atmosphere by MGC AnaeroPack-Jar and MGC AnaeroPack-Anaero, were purchased from Mitsubishi Gas Chemical (Tokyo, Japan). Blood agar plates (BAPs), BactoTM tryptic soy agar (TSA), and BactoTM tryptic soy broth (TSB) were purchased from Difco (Detroit, MI, USA).

### 4.2. Extraction and Isolation of Cembrene Diterpenoids from S. flexibilis Extract

**Figure 5:**
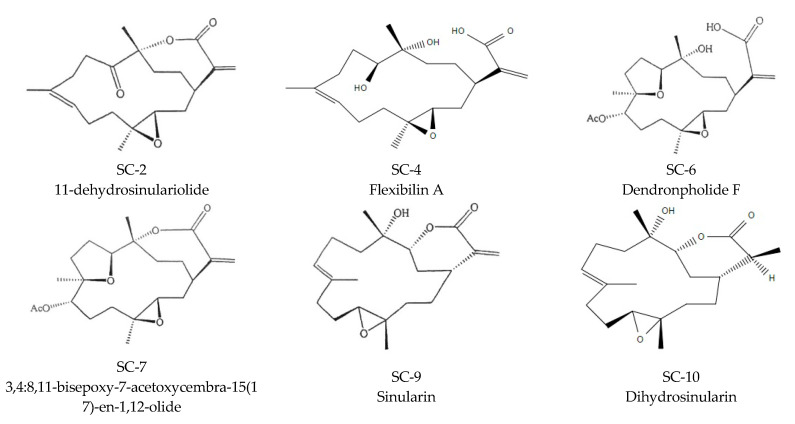
Structural formula of the six cembrene diterpenoids.

Soft coral *S. Flexibilis specimens were* initially collected off the coast of Pingtung, southern Taiwan and transferred to an 80-ton cultivating tank equipped with a flow-through sea water system in June 2005, and the material for this research work was collected from the tank in October 2015. A voucher specimen was deposited in the National Museum of Marine Biology and Aquarium, Taiwan. Freeze-dried and minced *S. flexibilis* (wet weight 3.8 kg, dry weight 420 g) was extracted exhaustively with EtOAc (2.0 L × 6) at room temperature. The EtOAc extract was evaporated under reduced pressure to obtain a residue (27.6 g), and the residue was subjected to column chromatography on silica gel using *n*-hexane, an *n*-hexane and EtOAc mixture of increasing polarity, and pure acetone to yield 13 fractions. Fraction 9, eluting with *n*-hexane-EtOAc (2:1), was further separated by silica gel open column chromatography with gradient elution (*n*-hexane-EtOAc, 2:1 to 1:1) to yield six subfractions (9A–F). Subfraction 9C was subjected to normal phase HPLC using *n*-hexane-EtOAc (2:1) to yield SC-2 and SC-7. Fraction 10, eluting with *n*-hexane-EtOAc (1:1), was further separated by silica gel open column chromatography with gradient elution (*n*-hexane-EtOAc, 1:1 to 1:2) to give nine subfractions (10A–I). Subfraction 10C was separated by normal phase HPLC using *n*-hexane-acetone (4:1) to yield SC-9 and SC-10. Fraction 11, eluting with *n*-hexane-EtOAc (1:2), was further separated by silica gel open column chromatography with gradient elution (*n*-hexane-EtOAc, 1:2 to 1:3) to yield four subfractions (11A–D). Subfraction 11D was further separated by normal-phase HPLC using *n*-hexane-acetone (2:1) to yield SC-4 and SC-6. (Figure 5). The fractions 9+10 (SF-E) was used for the further in vivo experiment.

### 4.3. Cutibacterium acnes-induced Ear Edema in Wistar Rats

**Figure 6:**
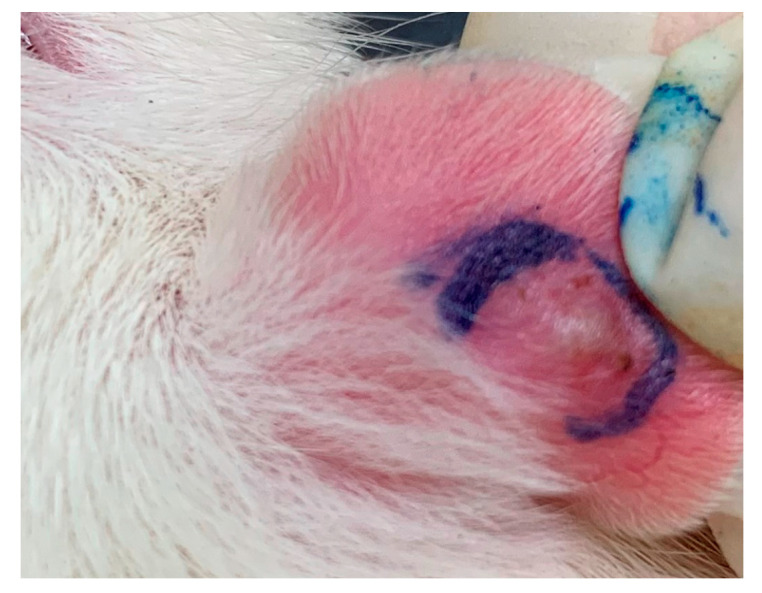
*C. acnes*-induced acne formation in Wistar rats.

The animal use protocol was reviewed and approved by the Institutional Animal Care and Use Committee (IACUC), Taipei Medical University (approval no. LAC-2019-009). All methods involved in the animal experiments were performed in accordance with previously established relevant guidelines and regulations. Male Wistar rats weighing 200 ± 10 g were bought from BioLASCO Taiwan (Yilan, Taiwan) and kept on a 12-h light/12-h dark cycle at 21 ± 2 °C with food and water *ad libitum*. *Cutibacterium acnes* (8 × 10^7^ colony-forming units (cfu)/20 μL) was intradermally injected into the left ear of rats to induce edema. Phosphate-buffered saline (PBS) was injected into the right ear as a vehicle control [3]. One day post-injection, the perimeter of the ear edema, including the diameter and height, was measured with calipers. When the volume of ear edema was calculated, the model was considered as being established successfully (Figure 6). SF-E ointment (5% and 10% SF-E in hydrophilic ointment) was applied (five rats per group) on the skin surface of the left ear. Anti-acne levels were observed daily as assessed by changes in edema. Experimental data were calculated as the difference between the measured value on the first day and that on the second, third, and fourth days.

### 4.4. Antibacterial Activities against C. acnes

*Cutibacterium acnes* (BCRC10723) was obtained from the Bioresource Collection and Research Center and cultured in BAP with an anaerobic atmosphere using the MGC AnaeroPack-Anaero and MGC AnaeroPack-Jar. Freshly grown *C. acnes* was diluted with BactoTM TSB, and 10 mL of prepared bacteria (4 × 10^8^ cfu/mL) was aseptically added to 90 mL of sterilized media (TSA) at 45 °C in a water bath. The seeded agar media were immediately mixed and poured into a petri dish. The six cembrene diterpenoids were tested against *C. acnes* by the diffusion method. Penicillin was used as the positive control. Plates were incubated under anaerobic conditions at 37 °C for 24 h, and inhibition zones were measured in millimeters. Each experiment was conducted in triplicates.

### 4.5. Anti-Inflammatory Activity against LPS-Treated RAW 264.7 Cells

Anti-inflammatory effects were analyzed in RAW 264.7 cells, which were cultured in DMEM with 10% FBS, 1% L-glutamine, and 1% penicillin-streptomycin, and maintained at 37 °C in a 5% CO_2_ atmosphere. RAW 264.7 cells (4.0 × 10^5^ cells/mL) were seeded in 96-well plates and co-treated with LPS (500 ng/mL) and test samples. After 24 h, NO production was determined by Griess reagent, and the absorption was detected at 530 nm. The percentage inhibition of NO production was presented as the anti-inflammatory activity. PGE_2_ concentrations in cell culture media were determined with a PGE_2_ enzyme-linked immunosorbent assay (ELISA) kit (Enzo Life Sciences, Farmingdale, NY, USA). The survival rate of RAW 264.7 cells was detected using an MTT assay.

Whole-cell lysates were prepared using radioimmunoprecipitation (RIPA) assay. Proteins in lysate samples (30 μg) were analyzed using 10% sodium dodecylsulfate-polyacrylamide gel electrophoresis (SDS-PAGE). Gels were transferred to nitrocellulose membranes and probed with primary iNOS (clone N-20), COX-2 (clone C-20), p-ERK (clone N-20), p-JNK (clone N-20), and glyceraldehyde-3-phosphate dehydrogenase (GAPDH; clone 6C5) antibodies (Santa Cruz Biotechnology, Santa Cruz, CA, USA). Membranes were visualized with a BCIP/NBT kit (Gibco BRL, Grand Island, NY, USA). Protein expression was quantified using the Azure Biosystem C300 Imaging System. GAPDH expression was used as an internal control to compare with protein expressions of iNOS, COX-2, p-ERK, and p-JNK [24].

### 4.6. Anti-Inflammatory Activity against Heat-Killed C. acnes-Treated RAW 264.7 and HaCaT Cells

*C. acnes* (4 × 10^8^ cfu/mL) was heated at 80 °C for 30 min and killed. Heat-killed *C. acnes* (HKC) was freeze-dried as a powder and maintained at 4 °C until use. RAW 264.7 and HaCaT cells were cultured in DMEM with 10% FBS, 1% penicillin-streptomycin, and 1% L-glutamine at 37 °C in a 5% CO_2_ atmosphere. RAW 264.7 cells (4 × 10^5^ cells/mL) and HaCaT cells (10^5^ cells/mL) were seeded in six-well plates and co-treated with HKC (300 μg/mL) and test samples [24]. After 24 h, NO production was determined by adding the Griess reagent in the cell culture medium, and the absorption was measured in an ELISA reader at 530 nm. The sample culture medium was removed, and RAW 264.7 cells and HaCaT cells were washed with PBS.

### 4.7. Anti-Proliferative Activity against Testosterone- and Dihydrotestosterone (DHT)-Treated HaCaT Cells

HaCaT cells (10^5^ cells/mL) were seeded in 96-well plates and co-treated with testosterone (5 μg/mL) or DHT (5 μg/mL) and test samples with no cytotoxicity for 48 h [2,25,26]. Cell proliferative activity was detected by an MTT assay.

### 4.8. Statistical Analysis

Data are presented as the mean and standard deviation (SD). Significance was calculated using Student’s *t*-test and a one-way analysis of variance (ANOVA) with SPSS software version 15 (SPSS, Chicago, IL, USA).

## 5. Conclusions

The main causes of acne include abnormal keratinization, abnormal sebum secretion, excessive commensal skin microbiota growth, and inflammatory reactions. In this study, the SF-E directly attenuated *C. acnes*-induced Wistar ear edema in vivo. In in vitro studies, we explored the antibacterial activity against *C. acnes*, inflammatory response of RAW 264.7 macrophages and HaCaT keratinocytes induced by LPS and HKC, and proliferation of HaCaT keratinocytes induced by DHT. We found three sinulariolides, 11-dehydrosinulariolide (SC-2), 3,4:8,11-bisepoxy-7-acetoxycembra-15(17)-en-1,12-olide (SC-7), and sinularin (SC-9) inhibited keratinocyte over-proliferation by DHT. SC-2, SC-7, and SC-9 also showed good anti-inflammatory abilities by inhibiting NO production through significantly inhibiting the activity of p-ERK and p-JNK by LPS and HKC. In particular, SC-9 was the most potent cembrene diterpenoid, presenting the best inhibitory effects on NO production and iNOS at 24 h, PGE_2_ at 6 h, and p-ERK and p-JNK at 30 min. Furthermore, SC-9 also inhibited keratinogenesis and sebum secretion at very low concentrations.

Taken together, we suggest that the SF-E and three cembrene diterpenoids (SC-2, SC-7, and SC-9) showed multiple anti-acne capabilities, including anti-keratinocyte proliferation, and anti-inflammatory actions. Therefore, the soft coral *S. flexibilis* has great potential to be developed as skin care products. It is hoped that in future, soft coral and their natural products can be developed into new generation anti-acne skin care products, or therapeutic agents with low side effects and expand the diversified development of marine natural products.

## Figures and Tables

**Table 1:** Cell viability, nitric oxide (NO) inhibition, 50 % inhibitory concentration (IC_50_) values for NO and prostaglandin E_2_ (PGE_2_) inhibition by six cembrene diterpenoids (SC) in lipopolysaccharide (LPS)- and heat-killed *Cutibacterium acnes* (HKC)-induced RAW 264.7 cells.

Sample	LPS-Induced RAW 264.7 Cells				HKC-Induced RAW 264.7 Cells
	Cell Viability (%) ^a^	NO Inhibition (%) ^a^	IC_50_ Value of NO Inhibition (μM)	IC_50_ Value of PGE_2_ Inhibition (μM) ^b^	IC_50_ Value of NO Inhibition (μM) ^c^
SC-2	102.3 ± 9.4	92.4 ± 3.0	5.66 ± 0.19	-	5.81 ± 0.4
SC-4	98.8 ± 1.2	−4.3 ± 1.6	-	-	-
SC-6	96.1 ± 0.4	−15.1 ± 0.0	-	-	-
SC-7	96.1 ± 8.4	50.2 ± 5.5	15.25 ± 0.25	-	7.42 ± 0.4
SC-9	98.9 ± 0.0	102.5 ± 1.4	3.85 ± 0.25	6.22	2.81 ± 0.2
SC-10	117.1 ± 1.9	28.2 ± 4.6	-	-	-

^a^ Experiments were carried out in LPS-induced RAW 264.7 cells for 24 h at 20 μM; ^b^ Experiments were carried out in LPS-induced RAW 264.7 cells for 6 h.; ^c^ Experiments were carried out in HKC -induced RAW 264.7 cells for 24 h.

**Table 2:** Anti-Proliferative effects of SC-2, SC-9, and SC-7 in testosterone- and dihydrotestosterone-treated HaCaT cells.

**Testosterone-Induced HaCaT Cells**					
**SC-2**		**SC-7**		**SC-9**	
**Conc.^a^ (μM)**	**Inhibition (%)**	**Conc.^a^ (μM)**	**Inhibition (%)**	**Conc.^a^ (μM)**	**Inhibition (%)**
1.25	11.9 ± 5.5	2.5	13.2 ± 0.7	0.08	6.5 ± 0.5
2.5	13.5 ± 1.1	5	14.0 ± 2.0	0.16	11.5 ± 1.3
5	17.4 ± 2.8	10	20.8 ± 0.9	0.32	19.1 ± 1.3
**Dihydrotestosterone-Induced HaCaT Cells**					
**SC-2**		**SC-7**		**SC-9**	
**Conc.^a^ (μM)**	**Inhibition (%)**	**Conc.^a^ (μM)**	**Inhibition (%)**	**Conc.^a^ (μM)**	**Inhibition (%)**
1.25	10.6 ± 3.0	2.5	9.5 ± 3.2	0.08	8.5 ± 0.7
2.5	12.3 ± 3.1	5	13.1 ± 2.9	0.16	16.0 ± 0.7
5	16.8 ± 4.0	10	13.8 ± 0.8	0.32	18.0 ± 3.1

^a^ Conc.: Concentration.
